# Leiomyomatosis Peritonealis Disseminata Following Laparoscopic Surgery With Uncontained Morcellation: 13 Cases From One Institution

**DOI:** 10.3389/fsurg.2021.788749

**Published:** 2021-12-09

**Authors:** Xin Chen, Haiyuan Liu, Honghui Shi, Qingbo Fan, Dawei Sun, Jinghe Lang

**Affiliations:** Department of Obstetrics and Gynecology, Peking Union Medical College Hospital, Chinese Academy of Medical Sciences & Peking Union Medical College, National Clinical Research Center for Obstetric & Gynecologic Diseases, Beijing, China

**Keywords:** clinical characteristics, disseminated peritoneal leiomyomatosis, iatrogenic, prognosis, treatment

## Abstract

**Objectives:** To investigate the clinical characteristics, treatment and prognosis of leiomyomatosis peritonealis disseminata (LPD) following laparoscopic surgery with uncontained morcellation and to summarize clinical features of iatrogenic LPD based on published literature together with our own experience.

**Methods:** A cohort of 13 cases with iatrogenic LPD diagnosed and treated in Peking Union Medical College Hospital from 2011 to 2020 was reported focusing on clinical characteristics, treatment and prognosis.

**Results:** All the patients had a history of laparoscopic myomectomy with uncontained morcellation. The average age was 35.6 (range 25–47) years. The interval between initial laparoscopic surgery and first diagnosis of LPD was 6.08 years on average (range 1–12). Most of the patients had no obvious symptoms. The accuracy of pre-operative diagnosis was low. Two patients had been treated with gonadotropin-releasing hormone agonist (GnRH-a) before surgery without obvious effect. The nodules of LPD are usually located in the lower half of the peritoneal cavity. The most commonly involved site was the pouch of Douglas. The number of nodules ranged from 3 to over 10, and they ranged in size ranged from 0.3 to 22 cm. All patients underwent surgical treatment: six patients underwent laparoscopy and seven underwent laparotomy. Pathology results confirmed LPD. The immunohistochemical profile indicated LPD tends to be positive strongly for desmin, caldesmon, ER, PR and SMA. Only one patient underwent post-operative treatment with GnRH-a. All patients were followed for an average period of 49 months without recurrence.

**Conclusion:** Iatrogenic LPD is a relatively rare condition. Patients usually exhibit no hormonal stimulation factors. Surgery is the main method of treatment, and hormone suppressive therapy is only rarely used. The nodules are usually large and less numerous, and most involve the pelvis. The prognosis of iatrogenic LPD seems good.

## Introduction

Leiomyomatosis peritonealis disseminata (LPD), also called disseminated peritoneal leiomyomatosis (DPL), is a rare gynecological disorder characterized by the dissemination of multiple smooth muscle nodules throughout the peritoneal surface. It was first described in 1952 by Wilson and Peale (1) and named by Taubert in 1965 (2). There have been ~200 cases of LPD reported in the literature (3). Iatrogenic LPD following laparoscopic surgery is a more rare condition, only recently recognized (4, 5). Approximately 28 papers covering 41 cases of iatrogenic LPD have been reported in English, most of them single case reports. Because of its rarity, the pathogenesis is poorly understood and proper management and prognosis have not been investigated very well. The present study, which has one of the largest cohorts of iatrogenic LPD, has focuses on clinical characteristics, treatment, and prognosis.

## Materials and Methods

From January 2011 to May 2020, seventeen cases of LPD were identified in Peking Union Medical College Hospital. Thirteen cases were iatrogenic LPD while four cases were spontaneous LPD without operation history. Our study only focused on iatrogenic LPD. The clinical data collected from medical records were retrospectively analyzed and evaluated. SPSS 21.0 statistical software was used for analysis. The study was exempted from review by the Human Investigation Review Board (IRB).

All patients were followed up regularly post-operatively with imaging. The plan of follow-up was that: once for 3 months for first year; once for 6 months for second year and once for 12 months for third year and afterward. Patients received gynecological ultrasound examination every 3 months and pelvic and abdominal MR image once a year.

## Results

### Clinical Manifestation

The clinical features of the 13 cases of LPD are summarized in Table 1. All patients had a history of laparoscopic myomectomy with uncontained morcellation conducted. No history of laparoscopic subtotal hysterectomy or laparoscopic myomectomy without morcellation was found. The average age at diagnosis was 35.6 years (range 25–47; median 37). The average gravidity was 1.85 (range 0–4; median 2) and the average parity was 0.77(range 0–2; median 1). The interval between initial laparoscopic surgery and first diagnosis of LPD was 6.08 years on average (range 1–12; median 6). The previous pathological results of four patients were unknown and the others were leiomyomas of the uterus. All patients denied the use of oral contraceptives or hormonal therapy. None of patients were pregnant at the time of diagnosis. Case 12 was a recurrent one, who developed LPD 1 year after laparoscopic myomectomy and recurred 3 years after total abdominal hysterectomy and LPD nodules.

**Table 1:** Clinical characteristics of 13 cases of iatrogenic LPD.

**Case**	**Age**	**G-P**	**History**	**Interval (years)**	**Previous pathology**	**Symptoms**	**Involvement sites**	**CA125 (U/ml)**	**Surgical procedure**	**Number of LPD nodules**	**Sizes of LPD nodules (cm)**	**Final pathology**	**GnRH-a**	**Follow-up (months)**
1	39	1–1	LM	5	NA	Asymptomatic	Douglas pouch, rectum	16.4	TAH+BS+MR	12	0.5–11	Leiomyoma	N	ANED (91)
2	37	0–0	LM LH	4	Leiomyoma	Asymptomatic	Right pelvic peritoneum, sigmoid mesocolon, adnexa, omentum, rectum	6.94	BSO+LND+Partial OMT+MR	>10	0.5–8	Leiomyoma	N	ANED (93)
3	32	2–1	LM	3	NA	Asymptomatic	Douglas pouch, pre-sacral region, subdiaphragm, omentum	42.3	LapMR+ Partial OMT	>10	1–10	Leiomyoma	N	ANED (80)
4	25	0–0	LM	3	Leiomyoma	Abdominal Pain	Sigmoid mesocolon, omentum, mesentery	70.8	MMR+Repair	>10	1–22	Leiomyoma	N	ANED (66)
5	30	2–1	LM	8	Leiomyoma	Asymptomatic	Appendix, Douglas pouch	36.7	LapMR	3	1–8	Leiomyoma	N	ANED (47)
6	40	4–2	LM	10	Leiomyoma	Asymptomatic	Omentum, bladder, Right Douglas pouch	23.3	LH+BS+MR	5	0.5–5	Leiomyoma	N	ANED (43)
7	47	3–0	LM	12	NA	Asymptomatic	Right retroperitoneum, Right lower abdominal peritoneum, Right colon serosal surface	15.3	LM+MR	3	0.3–2.5	Leiomyoma Adenomyosis	N	ANED (52)
8	45	3–1	LM	6	Leiomyoma	Abdominal mass	Bladder, mensentery	42.6	TAH+BS+MR	7	0.5–7.5	Leiomyoma	N	ANED (37)
9	26	0–0	TAM LM TAM	5	Leiomyoma	Abdominal discomfort	Abodiminal and Pelvic peritoneum, Mesentery, Sigmoid, Previous Trocar Site, Douglas pouch	77.4	TAM+MR	>10	0.2–3	Leiomyoma Adenomyosis	8 doses before surgery 6 doses after surgery	ANED (38)
10	26	0–0	LM^*^2	10	NA	Asymptomatic	Omentum, Small Intestine, Abdominal peritoneum	16.8	TAM+Partial OMT+MR	>10	0.5–1	Leiomyoma Adenomyosis	N	ANED (34)
11	45	2–1	LM	6	Leiomyoma	Asymptomatic	Douglas pouch, Sigmoid, Omentum	24.5	MR+OMT+MMR	3	0.5–8	Leiomyoma	N	ANED (26)
12	46	4–1	CS LM TAH	1.3	Cellular leiomyoma	Asymptomatic	Douglas pouch, pelvic peritoneum, Rectum	23.3	LapMR+BSO	4	0.4–3	Leiomyoma	N	ANED (18)
13	25	3–2	LM CS^*2^	9	Leiomyoma	Asymptomatic	Mesentery, Peritoneum, Rectus abdominis	81.7	LM+MR	3	0.5–15	Leiomyoma	6 doses before surgery	ANED (12)

*BS, bilateral salpingectomy; BSO, bilateral salpingo-oophorectomy; LH, laparoscopic hysterectomy; LM, laparoscopic myomectomy; MR, mass resection; MMR, mesenteric mass resection; OMT, omentectomy; TAH, total abdominal hysterectomy; TAM, total abdominal myomectomy; ANED, alive with no evidence of disease; AWD, alive with disease; NA, not available*.

**Figure 1:**
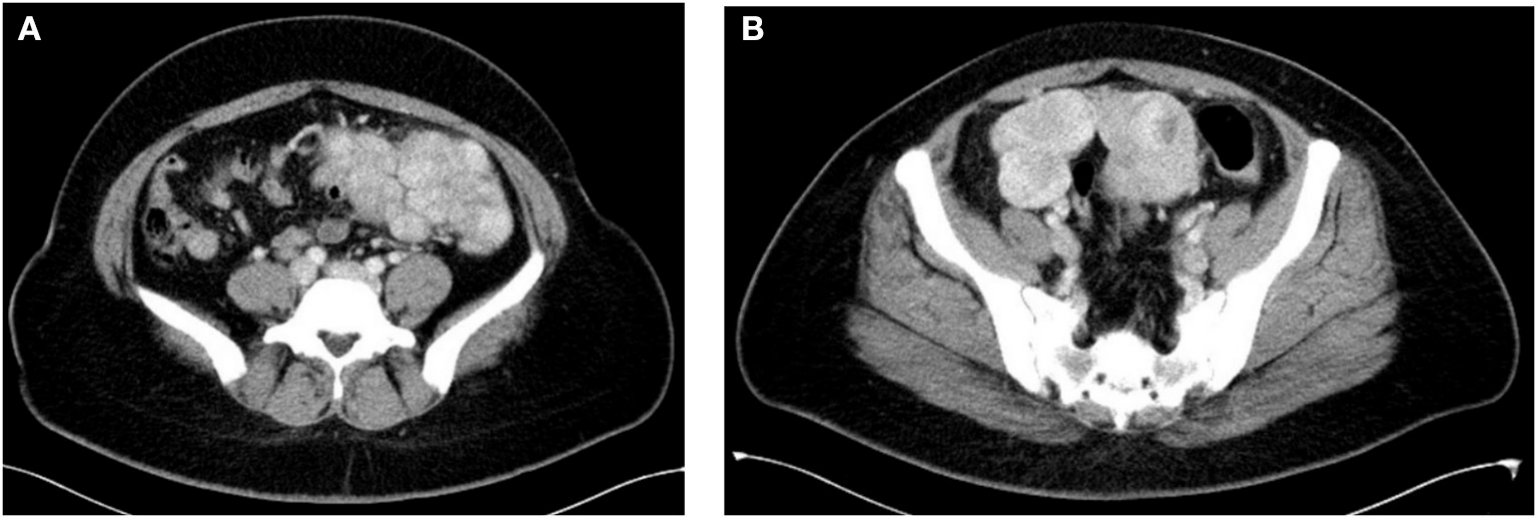
**(A,B)** Axial contrast enhanced CT showed multiple soft tissue lesions in mesentery with necrosis in some lesions (Case 4).

Eleven patients initially sought medical treatment because of an incidental finding of pelvic mass or leiomyoma recurrence observed during routine examination. Case 4 had acute lower abdominal pain accompanied by fever while waiting for the scheduled surgery. Computed tomography and MR imaging indicated that there was a necrosis of nodules (Figure 1). After antibiotic treatment, the abdominal pain relieved and the temperature returned to normal. Case 8 felt a mass in her lower abdomen herself and Case 9 had experienced abdominal discomfort for 6 months.

**Figure 2:**
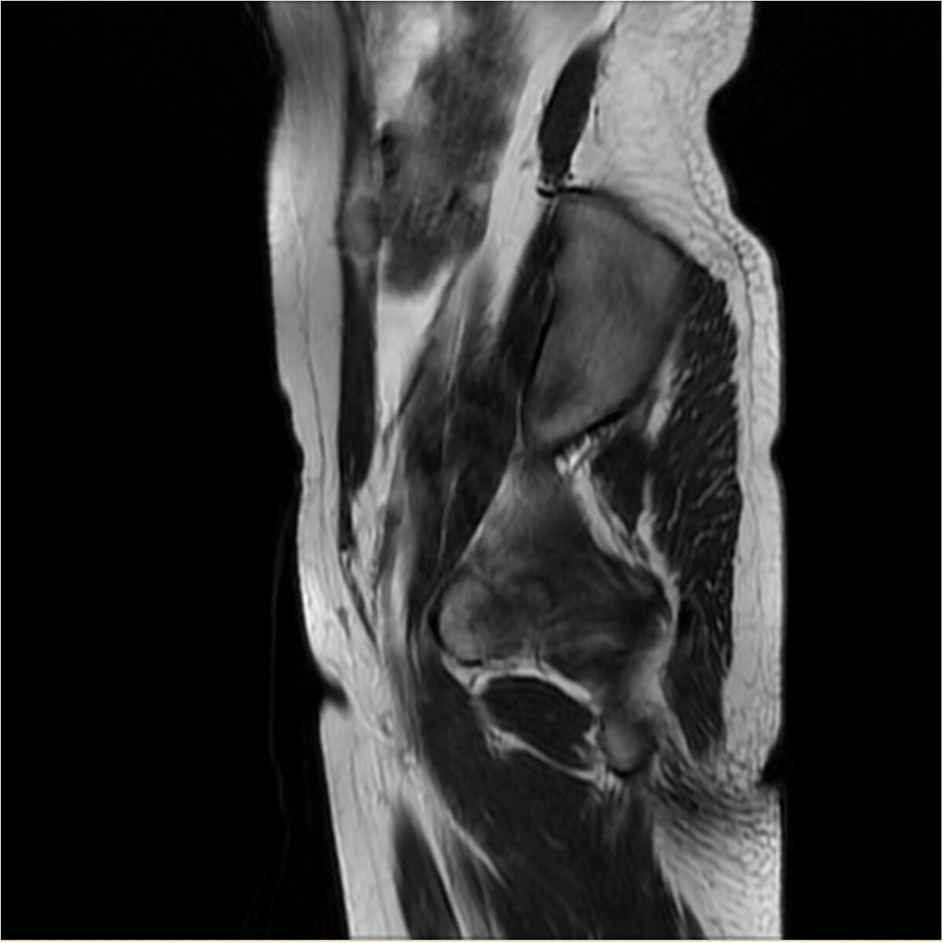
Sagittal MRI showed a nodule measuring 1.2 cm × 1.9 cm within the right rectus abdominis (Case 13).

The average serum CA125 level was 36.77 U/ml (range 6.9–81.7, median 24.5). Six patients had an elevated level of CA125. Eight patients underwent MRI examination before surgery. Four of them indicated multiple nodules in the pelvic and abdominal cavity. In Case 13, MRI showed a nodule on the abdominal wall (Figure 2).

The accuracy of pre-operative diagnosis was low. Four patients were suspected of malignant tumor before surgery while others were believed to have leiomyoma recurrence or broad ligament leiomyoma. Case 4 underwent CT-guided biopsy because of suspected malignancy. The pathological results of biopsy indicated leiomyoma.

With a diagnosis of uterus leiomyoma recurrence at that time, Cases 9 and 13 were treated with gonadotropin-releasing hormone agonist (GnRH-a) before surgery to decrease the size of uterine myomas. Case 9 had a slight decrease of uterine myomas after 8 injections of Enantone. In Case 13, the size of one nodule that was considered a subserous myoma increased from 10 cm to 13 cm after 6 injections of Enantone.

### Surgery

**Figure 3:**
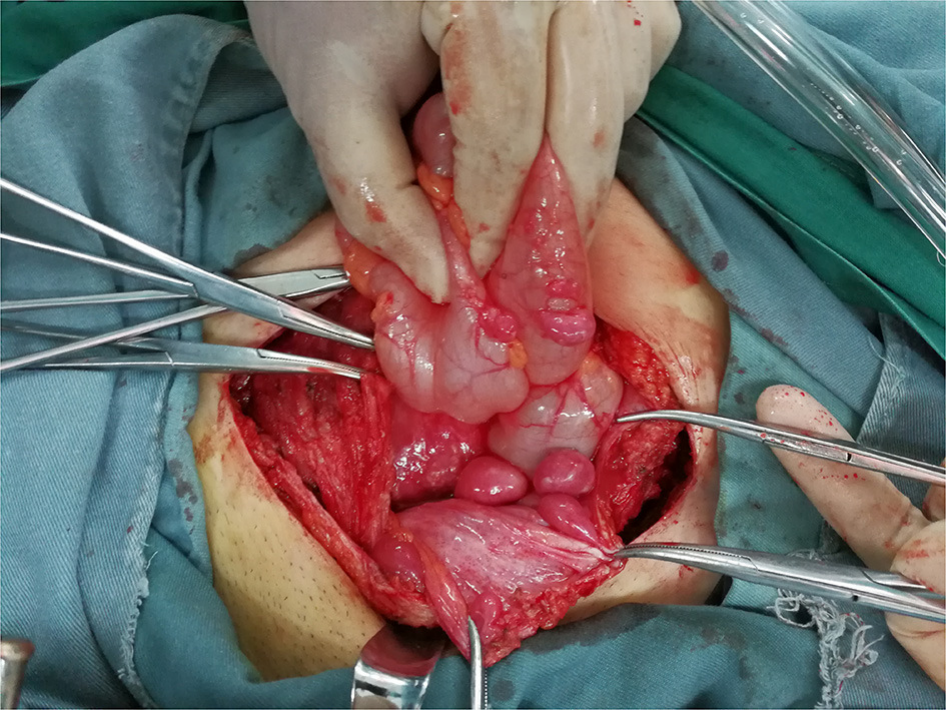
Multiple LPD nodules on the anterior peritoneum and the surface of colon (Case 9).

**Figure 4:**
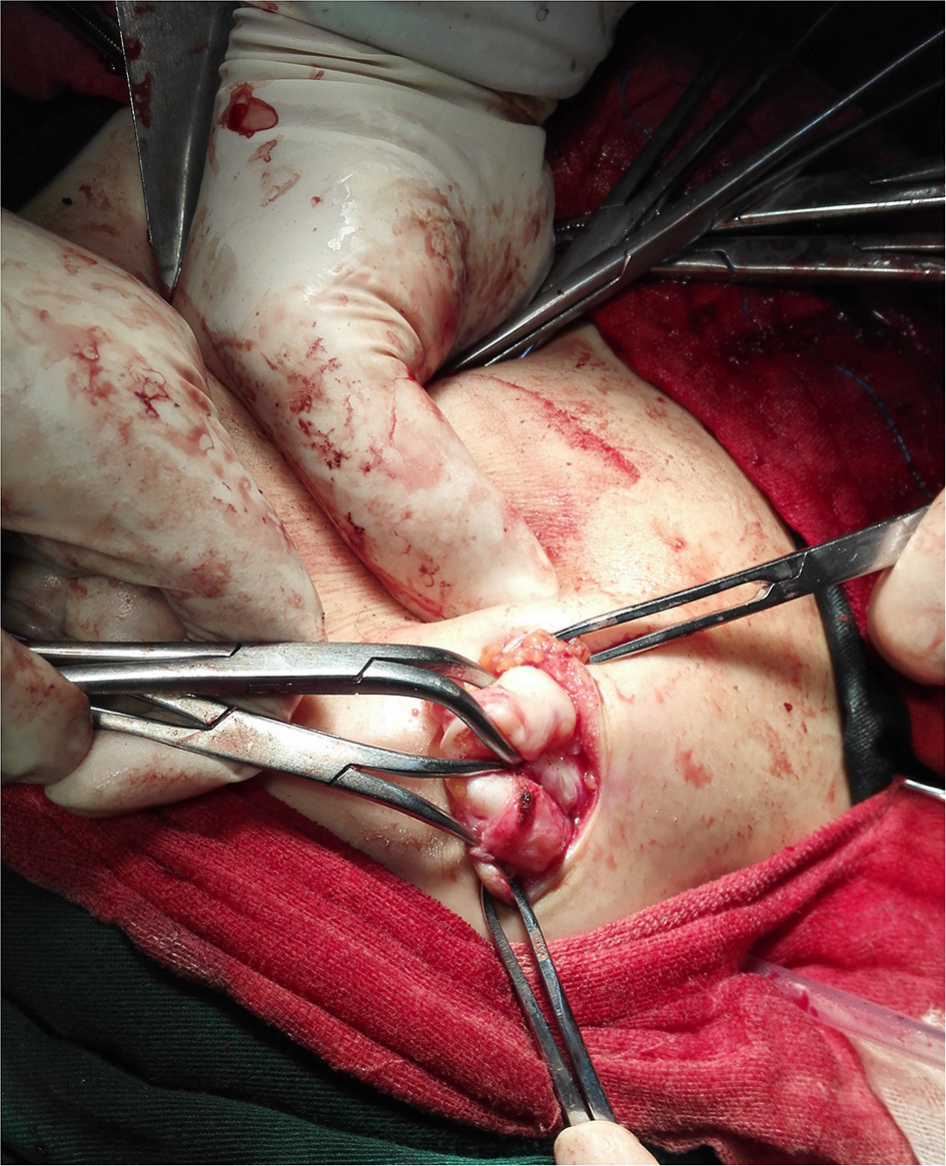
Intraoperative image of the subcutaneous nodule around the laparoscopy scar of previous myomectomy (Case 9).

During the operation, multiple nodules were found distributed in the abdominal and pelvic cavities. The number of the nodules ranged from 3 to over 10 and the size ranged from 0.3 to 22 cm. Nodules were most commonly found in the pouch of Douglas (seven cases), followed by pelvic and abdominal peritoneum (six cases) (Figure 3), omentum (six cases), sigmoid (four cases), rectum (three cases), abdominal wall (two cases) and surface of the bladder wall (two cases). One case each showed nodules in the adnexa, pre-sacral region, subdiaphragm, appendix, small intestine and surface of the right colon, respectively. The nodules involved the abdominal wall were near the laparoscopy scar of a previous myomectomy (Figure 4).

Only Case 9 showed coexisting ovarian endometriosis and the others had no evidence of pelvic endometriosis.

All patients received individualized surgical treatments tailored to their age, symptoms, and desire for fertility. The main purpose of the operation was the complete removal of all nodules. The surgical procedures included the following: 1. Total abdominal hysterectomy and LPD nodule resection (Cases 1 and 8). 2. Abdominal LPD nodule resection (Cases 2, 4, and 11). Case 2 underwent removal of both adnexa, omentum and right pelvic lymph nodes at the same time. Case 4 underwent repair because of the damage to the serosal surface of small intestine. 3. Laparoscopic removal of LPD nodules (Cases 3, 5, and 12). Appendectomy was performed in Case 5 and bilateral adnexectomy in Case 12. 4. Laparoscopic removal of the uterus and LPD nodules (Case 6). 5. Laparoscopic myomectomy and removal of LPD nodules (Case 7). 6. Abdominal myomectomy and LPD nodule resection (Cases 9 and 10). All patients had uncomplicated post-operative course.

### Pathology

On examination, the nodules were usually found to be round, firm, and well-defined. In some cases, the nodules had fused into a large mass. In Cases 7, 9, and 10, one nodule had cystic cavities with hemorrhage on the cut section.

On microscopic examination, all specimens showed features typical of uterine leiomyoma, including proliferation of interlacing bundles of spindle cells without mitotic figures or cell atypia, or tumor cell necrosis. Histopathologic analysis in combination with the position of the detected nodules and the patients' history confirmed the diagnosis of LPD.

**Figure 5:**
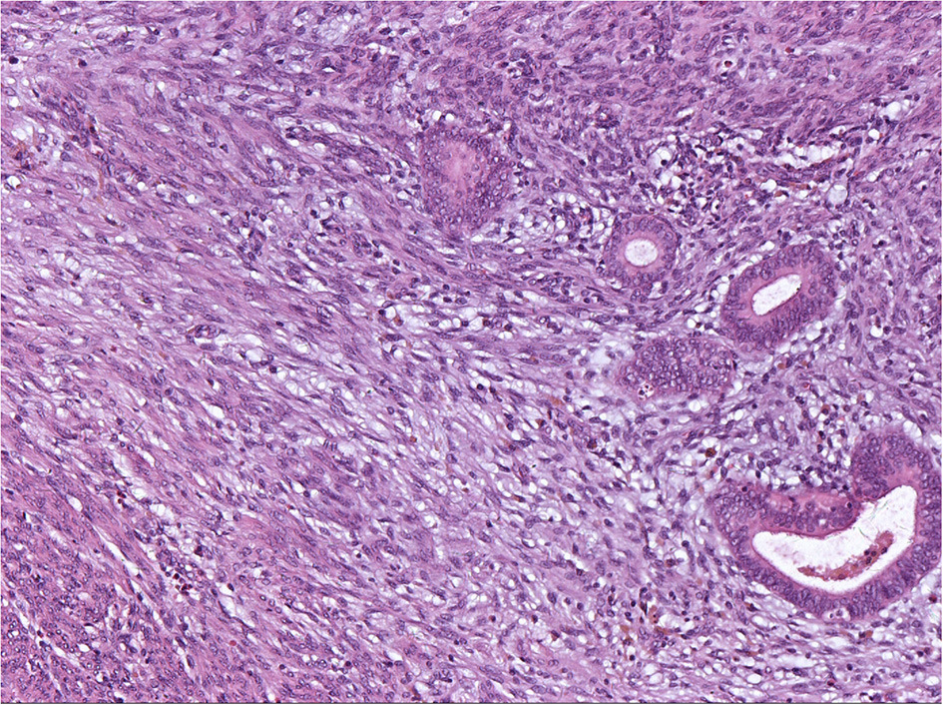
Endometrial glandular epithelium and stroma embedded in hyperplastic smooth muscle. HE × 100 (Case 7).

The histopathologic analysis of some nodules from Cases 7, 9, and 10 showed these LPD nodules to be adenomyoma because the endometrial glands and stroma were present within the spindle cells (Figure 5).

Of 13 patients, 8 underwent immunohistochemical staining. Their profiles are summarized in Table 2. The data indicated that LPD tends to be strongly positive strongly for desmin, caldesmon, ER, PR, and SMA (smooth muscle actin), but negative for S-100, CD10, CD117, and CD34. Six cases (Cases 3, 4, 5, 6, 8, and 13) were labeled for Ki-67 and they showed a low proliferation-index (10, 2, 2, 3, 5, and 1%, respectively).

**Table 2:** Immunohistochemical staining profile.

**Antigen**	**Cases (number+/number tested)**	**Positive rate,%**
Desmin	8/8	100%
Caldesmon	3/3	100%
ER	5/5	100%
PR	5/5	100%
S-100	0/4	0%
CD10	0/4	0%
CD117	0/3	0%
CD34	0/5	0%
SMA	5/5	100%
AE1/AE3	0/2	0%
P53	1/3	33.30%

### Follow-Up

Only Case 9 underwent post-operative therapy. She received six injections of GnRH-a to prevent recurrence. All patients were on regular follow-up through ultrasound examination and MR image. As of May 2021, the average follow-up period was 49 months and no recurrence was observed.

Case 9 and Case 10 had a desire for fertility. During follow-up, both patients were successfully pregnant and delivered by Cesarean section. No LPD nodules were found during the period of gestation or Cesarean section surgery.

## Discussion

In this study, the surgical history of all the patients is laparoscopic myomectomy with uncontained morcellation. Morcellation can help fragment large specimen into small pieces granting removal through a small incision, which makes laparoscopic surgery possible. But the malignant and benign spreads of morcellated tissue have attracted more and more attention (5, 6). Iatrogenic LPD is one of benign complications of uncontained morcellation. It is estimated that the overall incidence of LPD after laparoscopic uncontained morcellation was 0.12–0.95% (5, 6). In our institution, there were ~12,200 laparoscopic myomectomy surgeries from 2011 to 2020 and only 13 cases of iatrogenic LPD were identified. Therefore, the iatrogenic LPD is a rare condition as is reported in the literature.

The etiology and pathophysiology of LPD remains unclear. Hormonal theory is the classical hypothesis for spontaneous LPD because it occurs mainly in the reproductive-aged females, and some cases are found after use of oral contraceptives and hormonal replacement therapy, or during pregnancy and in the presence of an estrogen-secreting tumor (7, 8). In our case series, LPD are not associated with pregnancy. No patients were found LPD nodules during pregnancy. Case 9 and Case 10 were successfully pregnant and delivered during follow-up. No disseminated nodules were found during the period of gestation or Cesarean section surgery. Case 13 developed LPD after completing 2 pregnancies. In literature, only a 35-year-old female patient had an early intrauterine gestation but seeding tumors had been found before the pregnancy began (9). One patient with iatrogenic LPD had been intermittently taking oral contraceptive pills before diagnosis (10). Ovarian granulosa cell tumors are not found in all iatrogeinc LPD (4). It suggests that hormone stimulation may be not crucial to the development of iatrogenic LPD.

The pre-operative diagnosis of LPD is challenging. Most of the patients had no obvious symptoms. Its lack of specific characteristics causes a very high rate of pre-operative misdiagnosis. In our case series, some cases were suspected of malignant tumors because imaging examination showed multiple lesions in the abdominal cavity. In suspected patients, image-guided FNA is a good choice to exclude the probability of malignancy and avoid inappropriate surgical treatment (11–14). Unclear diagnosis makes it impossible to perform pre-operative treatment. Kumar et al. reported one case of iatrogenic LPD diagnosed by an US-guided biopsy and received a single dose of GnRH-a, which resulted in a small decrease in the size of the mass (11). Two patients in our study underwent pre-operative GnRH-a treatment with similar effect.

In our cases, the sites of involvement are concentrated mainly in the areas below the umbilicus, in accordance with the movement of tissue fragments to the lower part of the abdomen because of gravity. The LPD nodules of Cases 7 and 13 were mainly located on the right side of the abdominal and pelvic cavity. This may be because the morcellator is usually placed on the right side in our institution. The nodules of iatrogenic LPD are usually large in size and less numerous, which differs from the carcinomatosis-like dissemination of typical LPD (4). An iatrogenic nodule as large as 34 cm in diameter has been reported (12).

There is no consensus regarding the optimal treatment for LPD. Spontaneous regression of disseminated nodules has never been reported in iatrogenic LPD (4), so surgery is the treatment of choice. The scope of surgery should be personalized according to the patient's age, symptoms, fertility requirements, and previous treatment history. There is also no consensus regarding whether laparotomy or laparoscopy is more suitable. During laparoscopic removal of LPD nodules, the use of morcellation may leads to re-dissemination. In our study, six patients underwent laparoscopic surgery. In Case 9, a confined containment bag with a power morcellation was used to remove the uterine leiomyomas and disseminated nodules. In Case 13, the nodules were removed through an enlarged umbilical incision with the help of wound protector. These two methods can help reduce the risk of re-dissemination (15).

LPD coexisting with endometriosis within the same lesion has been occasionally reported. Toriyama et al. thought the presence of endometrial tissues within LPD lesions supports the hypothesis of submesothelial multipotential stem cells (16). In that study, none of the cases was iatrogenic LPD. Iatrogenic endometriosis and adenomyosis following laparoscopic hysterectomy have been described in previous works (17). In our study, nodules of the adenomyoma are most likely to come from the co-implantation of endometrium and smooth muscle.

Hormonal suppressive therapy is seldom used after surgery for iatrogenic LPD with only six cases reported (18–23). One had recurrence 2 years later (23) and the follow-up period of the remaining cases was too short for meaningful evaluation of therapeutic effects. Chemotherapy is only reported in the cases of LPD with malignant transformation, and the agents include doxorubicin, cyclophosphamide, cisplatin, ifosfamide, etoposide, etc. (24).

Because of its rarity, it is not clear which factors are related to recurrence of iatrogenic LPD. In our data, only one case experienced recurrence of LPD but no evidence of relapse after treatment in our institution. In the literature, the longest follow-up was 96 months, and there was no recurrence (8). No malignant transformation of iatrogenic LPD has been reported but long-time follow-up is undoubtedly needed.

In addition to laparoscopic surgery, a case of LPD in a patient with a history of hysteroscopic myomectomy has also been reported (25). The pathophysiology of LPD needs further study. The limitation of this study is its retrospective nature, the small number of cases and the monocentric setting. At the same time, the difficulty of diagnosis may lead to undetection of several cases.

## Conclusion

Based on published reports and series and on our own experience, we summarize some features of iatrogenic LPD. First, patients usually exhibit no hormonal stimulation factors, such as contraceptive pills, pregnancy, or estrogen-secreting tumors. Second, the effectiveness of hormone suppressive therapy in the treatment of iatrogenic LPD remains to be evaluated. Spontaneous regression of disseminated nodules has never been reported. So surgical resection is main choice of treatment. Third, the nodules of iatrogenic LPD, which are usually large and less numerous, are mainly located in the lower half of the peritoneal cavity below the level of the umbilicus, with the greatest concentration in the pelvis. Finally, from our experience, iatrogenic LPD usually has favorable prognosis but a regular follow-up is quite necessary.

## Data Availability Statement

The original contributions presented in the study are included in the article/supplementary material, further inquiries can be directed to the corresponding author/s.

## Ethics Statement

Written informed consent was obtained. The study was exempted from review by the Human Investigation Review Board (IRB).

## Author Contributions

HL, HS, QF, DS, and JL diagnosed the patients. XC wrote the manuscript. All authors revised the manuscript.

## Conflict of Interest

The authors declare that the research was conducted in the absence of any commercial or financial relationships that could be construed as a potential conflict of interest.

## Publisher's Note

All claims expressed in this article are solely those of the authors and do not necessarily represent those of their affiliated organizations, or those of the publisher, the editors and the reviewers. Any product that may be evaluated in this article, or claim that may be made by its manufacturer, is not guaranteed or endorsed by the publisher.
